# Wie gut ist das Wissen über sexuell übertragbare Infektionen in Deutschland? Ergebnisse der ersten bundesweiten repräsentativen Befragung zu Gesundheit und Sexualität (GeSiD)

**DOI:** 10.1007/s00103-021-03319-8

**Published:** 2021-04-21

**Authors:** Silja Matthiesen, Ursula von Rüden, Arne Dekker, Peer Briken, Susanne Cerwenka, Claudia Fedorowicz, Christian Wiessner

**Affiliations:** 1 Institut für Sexualforschung, Sexualmedizin und Forensische Psychiatrie, Universitätsklinikum Hamburg-Eppendorf, Martinistr. 52, 20246 Hamburg, Deutschland; 2 Referat Forschung, Qualitätssicherung, Bundeszentrale für gesundheitliche Aufklärung (BZgA), Köln, Deutschland; 3 Institut für Medizinische Biometrie und Epidemiologie, Universitätsklinikum Hamburg-Eppendorf, Hamburg, Deutschland

**Keywords:** Erwachsenensexualität, GeSiD-Studie, Sexuelle Gesundheit, Sex-Survey-Forschung, Wissen über sexuell übertragbare Infektionen (STI), Adult sexuality, GeSiD study, Sexual health, Sex survey research, Knowledge about sexually transmitted infections (STIs)

## Abstract

**Hintergrund:**

Sexuell übertragbare Infektionen (STI) sind ein relevanter Risikofaktor für die sexuelle Gesundheit des Einzelnen und der Bevölkerung. Für eine zielgruppenspezifische Präventionsarbeit ist der Wissensstand zu STI in verschiedenen Bevölkerungsgruppen daher von besonderem Interesse.

**Ziel:**

Ziel der vorliegenden Arbeit ist es, den Wissensstand zu neun STI in der deutschen Bevölkerung zu analysieren. Dafür werden Zusammenhänge mit soziodemografischen Variablen, sexualitätsbezogenen Merkmalen sowie der subjektiven Zufriedenheit mit dem Informationsstand untersucht.

**Methode:**

Die GeSiD-Studie „Gesundheit und Sexualität in Deutschland“ erhob von 4955 Personen per Face-to-Face-Interview repräsentative quantitative Daten zum Wissensstand zu STI. Als Auswahlverfahren wurde eine zweifache Zufallsstichprobe gezogen. Dafür wurden zunächst 200 *Sample Points* (Gemeinden) in ganz Deutschland regional proportional ausgewählt. Anschließend wurde eine Zufallsstichprobe von Adressen über die jeweiligen Einwohnermeldeämter gezogen. Die Teilnahmequote betrug 30,2 %; das Durchschnittsalter lag bei 46,3 Jahren.

**Ergebnisse:**

Wissen über HIV/Aids war in allen Altersgruppen weit verbreitet. Andere STI waren deutlich weniger bekannt. Besonders wenig informiert zeigten sich Ältere und Befragte mit niedrigem Bildungsstand, regionaler sozialer Benachteiligung sowie mit Migrationshintergrund. Eine höhere Anzahl von SexualpartnerInnen hing mit einem besseren Wissensstand zusammen. Gut informiert zeigten sich Personen, die sich nicht als heterosexuell beschreiben, sowie Personen, die schon einmal an einer STI erkrankt waren.

**Fazit:**

Heterosexuelle Erwachsene in Deutschland sind unzureichend über STI informiert. Zielgruppenspezifische Anstrengungen zur Verbesserung des Wissens über STI sind nötig, um sexuelles Risikoverhalten zu vermindern und die Inanspruchnahme von Präventionsangeboten zu verbessern.

## Abstract (en)

**Background:**

Sexually transmitted infections (STIs) are a relevant risk factor for the sexual health of individuals and the population. Therefore, the level of awareness and knowledge about STIs in different population groups is of particular interest for specific prevention work.

**Objectives:**

The aim of this study was to analyze the awareness of and knowledge about nine STIs in the German population. To do so, the study examined correlations with sociodemographic variables, sexuality-related characteristics, and subjective satisfaction with the level of information.

**Methods:**

The GeSiD study “German Health and Sexuality Survey” collected representative quantitative data from 4955 persons via face-to-face interviews on the state of knowledge about STIs. As a selection procedure, a two-step random sample was collected. Firstly, 200 sample points (municipalities) were initially selected proportionally across Germany. Secondly, a random sample of addresses was drawn from the respective residents’ registration offices. The participation rate was 30.2%; the average age was 46.3 years.

**Results:**

Knowledge about HIV/AIDS was widespread in all age groups, but other STIs were significantly less known. Older people and respondents with a low level of education were particularly poorly informed. Local social disadvantage and a family history of migration were also negatively correlated with knowledge about STIs. A higher number of sexual partners is related to a better level of knowledge. In addition, persons who do not describe their sexual orientation as heterosexual as well as those with a history of STIs were well informed.

**Conclusion:**

Heterosexual adults in Germany are insufficiently informed about the risks of STIs. Therefore, target-group-specific efforts are needed to improve knowledge about STIs in order to reduce sexual risk behavior and improve the utilization of prevention programs among socially disadvantaged groups.

## Einleitung

Sexuell übertragbare Infektionen (STI) sind weltweit ein ernstes Problem mit schätzungsweise einer Million Neuinfektionen pro Tag [1]. STI können ein breites Spektrum negativer Folgen für die individuelle sexuelle Gesundheit haben, von körperlichen Beschwerden bis zur Unfruchtbarkeit, schweren Komplikationen in der Schwangerschaft und Schädigungen des Fötus bis hin zum Verlust des Lebens der Betroffenen. Die weltweit häufigsten viralen STI-Erreger sind das humane Papillomavirus (HPV), Herpes-Simplex-Virus 2 (HSV-2), humanes Immundefizienzvirus (HIV) und Hepatitis B; die am weitesten verbreiteten bakteriellen STI sind Chlamydien, Gonorrhö und Syphilis [2]. Die Weltgesundheitsorganisation (WHO) hat im Jahr 2016 die „Global Health Sector Strategy on Sexually Transmitted Infections, 2016–2021“ initiiert, um weltweit einen Rückgang der STI zu fördern [3].

Für Deutschland entwickelten – basierend auf einem Beschluss des Bundeskabinetts aus dem Jahr 2016 – das Bundesministerium für Gesundheit und das Bundesministerium für wirtschaftliche Zusammenarbeit und Entwicklung gemeinsam eine Strategie, die bis zum Jahr 2030 die Eindämmung von HIV, Hepatitis B und anderen STI zum Ziel hat [4]. Im Mittelpunkt hierbei steht, „Wissen zu vermitteln und Kompetenzen zu erweitern, um die Voraussetzungen dafür zu schaffen, dass Menschen verantwortungsvoll mit sexuell übertragbaren Infektionen umgehen und darin bestärkt werden, Präventions- und Versorgungsangebote wahrzunehmen“ [4].

Hintergrund der Strategie ist, dass es bislang trotz vielfältiger Maßnahmen^1^ nicht gelungen ist, in der bundesdeutschen Öffentlichkeit das gleiche Bewusstsein für die verschiedenen STI zu schaffen, wie es bezogen auf HIV/erworbenes Immunschwächesyndrom (HIV/Aids) der Fall ist. Die seit Ende der 1980er-Jahre jährlich durchgeführte Repräsentativbefragung *Aids im öffentlichen Bewusstsein der Bundesrepublik Deutschland* zeigt, dass die Aufklärung zu anderen STI in der Schule deutlich hinter der zu HIV/Aids zurückfällt [5]. Dementsprechend ist der Wissensstand zu STI in der Bevölkerung insgesamt immer noch relativ niedrig. Bei den im Rahmen der Studie abgefragten sieben STI schwankt die Bekanntheit stark: Fast die Hälfte der über 16-jährigen Allgemeinbevölkerung kennt im Jahr 2016 Syphilis (49 %) und Gonorrhö/Tripper (48 %). Die Bekanntheit von Chlamydien ist in den letzten Jahren auf 14 % gestiegen; sie lag vor 10 Jahren noch bei 1 %. Deutlich weniger Befragte geben Hepatitis und Herpes als bekannt an (jeweils 12 %). Kondylome bzw. Feigwarzen und Trichomoniasis werden noch seltener genannt (6 %/1 %; [5]).

Abgesehen von den Daten aus *Aids im öffentlichen Bewusstsein der Bundesrepublik Deutschland* liegen zum Wissensstand zu STI in Deutschland quantitative Studien bislang ausschließlich mit Jugendlichen (SchülerInnen) vor [6–8]. Hierzu wurden 2011 in Bremen und Bremerhaven und 2012 in Berlin jeweils Befragungen von mehr als 1000 SchülerInnen durchgeführt. Beide Studien kamen zu dem Ergebnis, dass der Wissensstand von Jugendlichen zu den verschiedenen STI ungenügend ist und dringend verbessert werden müsste. Die AutorInnen der Berliner Studie schlussfolgerten pointiert: „Unsere Ergebnisse zeigen, dass bezogen auf die meisten STI selbst Gruppen, die in der Regel nachweislich über bessere Kenntnisse im Bereich der sexuellen Gesundheit verfügen (wie weibliche Jugendliche, akademisch hochgebildete Personen und SchülerInnen aus nichtethnischen Minderheiten), nur unbefriedigende Kenntnisse haben“ (eigene Übersetzung; [6]).

Nur aus wenigen anderen europäischen Ländern liegen aktuelle Daten zum Wissensstand zu STI vor. Diese Studien beschäftigen sich allerdings hauptsächlich mit dem Wissenstand zu HPV und einer HPV-Impfung^2^ bei Jugendlichen und jungen Erwachsenen: So untersuchten zuletzt Coccino et al. [9] den Kenntnisstand zu HPV bei 9988 italienischen UniversitätsstudentInnen. Patel et al. [10] untersuchten den Wissensstand lettischer Jugendlicher zu HPV, nachdem sie schon 2015 ein systematisches Review zum Wissensstand von Jugendlichen zu HPV aus verschiedenen europäischen Ländern vorgelegt hatten [11]. Das letzte systematische Review, das den Wissensstand von Jugendlichen zu verschiedenen STI und Studien aus mehreren Ländern einbezieht, stammt aus dem Jahr 2011 [12]. Schon damals kamen die AutorInnen zu dem Ergebnis, dass die einbezogenen 15 Studien lediglich „low levels of awareness and knowledge of sexually transmitted diseases, with the exception of HIV/Aids“ berichteten.

Bezogen auf die Erwachsenenbevölkerung liefert die britische Studie *National Survey of Sexual Attitudes and Lifestyles* (Natsal-3) mit mehr als 15.000 Befragten die fundiertesten und umfangreichsten Analysen zum Vorkommen und der Verbreitung von STI in Großbritannien (vgl. www.natsal.ac.uk/publications). Die Natsal-Studien sind ein wegweisendes Beispiel für die Untersuchung von STI, weil es dort gelungen ist, neben Selbstauskünften zu den bisherigen Erkrankungen auch Speichel- und Urinproben zu entnehmen und damit objektive Messdaten zum Vorkommen bestimmter STI in der Allgemeinbevölkerung zu erhalten. Darüber hinaus ermöglicht es die Größe der Stichprobe, viele verschiedene Facetten der Prävention und Behandlung von STI zu untersuchen [13–17].

Bislang gab es für Deutschland keine bevölkerungsrepräsentativen Surveys, die das Sexualverhalten und die sexuelle Gesundheit der Erwachsenenbevölkerung umfassend abbildeten. Mit der GeSiD-Studie zu „Gesundheit und Sexualität in Deutschland“ ist es nun gelungen, erstmalig repräsentative Daten zum Sexualverhalten und zum Wissen über STI in der Altersgruppe der 18- bis 75-Jährigen zu erheben (für weitere Informationen zur Studie: www.gesid.eu).

Ziel der vorliegenden Arbeit ist es, den Wissensstand zu neun verschiedenen, in der Bevölkerung oder bestimmten Teilgruppen besonders verbreiteten STI zu analysieren: HIV/Aids, Syphilis, Gonorrhö/Tripper, Hepatitis B, genitaler Herpes, Filzläuse, Chlamydien, Genitalwarzen und Trichomoniasis.^3^

Detaillierte und krankheitsspezifische Kenntnisse über STI sind die Voraussetzung für den Schutz vor einer Erkrankung. Daher wurden folgende Aspekte untersucht:

1. Bekanntheit der verschiedenen STI,
2. Einfluss soziodemografischer Faktoren (Alter, Bildungsstand, Muttersprache, regionale soziale Deprivation),
3. Einfluss sexualitätsbezogener Faktoren (sexuelle Orientierung, Anzahl von SexualpartnerInnen, STI in der Vorgeschichte),
4. subjektive Zufriedenheit mit dem eigenen Kenntnisstand zu verschiedenen STI.

## Daten und Methodik

### Studie und Datenerhebung

Zwischen 2016 und 2020 wurden am Institut für Sexualforschung, Sexualmedizin und Forensische Psychiatrie des Universitätsklinikums Hamburg-Eppendorf zunächst eine Pilotstudie [18] und dann die bundesweite Bevölkerungsstudie „Gesundheit und Sexualität in Deutschland – GeSiD“ durchgeführt. Das Forschungsprojekt wurde von der Bundeszentrale für gesundheitliche Aufklärung (BZgA) gefördert, die Datenerhebung erfolgte in Zusammenarbeit mit dem Sozialforschungsinstitut Kantar.

Das Befragungsgebiet der GeSiD-Studie ist die Bundesrepublik Deutschland; die Grundgesamtheit sind deutschsprachige Personen in Privathaushalten im Alter von 18–75 Jahren. Als Auswahlverfahren wurde eine zweifach geschichtete Zufallsstichprobe erhoben. Dafür wurden im ersten Schritt zunächst 200 Sample Points (Gemeinden) in ganz Deutschland ausgewählt, die regional so gestreut waren, dass sowohl städtische als auch ländliche Regionen, Groß- und Kleinstädte und nord-, süd-, ost- und westdeutsche Gemeinden angemessen vertreten waren. Anschließend wurde im zweiten Schritt eine Zufallsstichprobe von Namen und Adressen über die jeweiligen Einwohnermeldeämter gezogen. Dies stellt ein qualitativ besonders hochwertiges methodisches Verfahren dar, um ein möglichst genaues Abbild der in Deutschland lebenden Bevölkerung zu erreichen.

Alle Teilnehmenden unterschrieben vor Beginn des Interviews eine Einwilligungserklärung, die sie über die Ziele der Studie, die Anonymisierung und den Datenschutz sowie die Freiwilligkeit der Teilnahme aufgeklärte. Die persönlichen Interviews wurden bei den Befragten zu Hause computergestützt durchgeführt (CAPI, Computer Assisted Personal Interview), wobei ein umfangreicher Teil des Fragebogens als Selbstausfüllerteil (CASI, Computer Assisted Self Interview) vorgesehen war. Nach Abschluss des Interviews erhielten die Teilnehmenden eine Aufwandsentschädigung von 30 €. Frauen wurden von Frauen und Männer von Männern befragt.

Der Fragebogen wurde auf der Basis einer ausführlichen Recherche der internationalen Vergleichsstudien entwickelt und im Rahmen einer Pilotstudie an 1155 Personen getestet [19]. Er umfasst mehr als 260 Fragen zu verschiedenen sexualitätsbezogenen Themen und schließt an die WHO-Definition von sexueller Gesundheit an [20]. Die durchschnittliche Dauer der Interviews lag bei 51 min; je nach Umfang der bisherigen Sexual- und Partnerschaftserfahrungen variierte die Interviewdauer zwischen 19 min und 208 min.^4^

### Stichprobe

Die untersuchte Stichprobe umfasst 4955 Personen, davon 2336 Männer und 2619 Frauen. Um eine detaillierte Untersuchung des sexuellen Verhaltens in der Altersgruppe mit dem höchsten Risiko für STI zu ermöglichen, wurden jüngere Altersgruppen übererfasst (Oversampling): Männer und Frauen im Alter von 18–35 Jahren machen 37,7 % (*n* = 1869) der Stichprobe aus. Die Teilnahmequote wurde nach AAPOR-Kriterien (American Association for Public Opinion Research) berechnet [21] und beträgt 30,2 % (AAPOR Response Rate 4). Die GeSiD-Stichprobe zeigte sich im Vergleich zum Mikrozensus 2018 weitgehend repräsentativ für die deutsche Bevölkerung, allerdings waren Männer und Personen mit niedrigem Bildungsniveau leicht unterrepräsentiert. Daher wurden die Daten für die weiteren Analysen bezogen auf Geschlecht, Alter, Bildung, Nationalität und Region statistisch gewichtet. Eine ausführliche Beschreibung von Studiendesign, Datenerhebung, Stichprobe, Gewichtung und Repräsentativität findet sich bei Matthiesen et al. 2021 (für nähere Informationen zur Methodik siehe [22] und www.gesid.eu).

### Untersuchte Variablen

Im Rahmen der GeSiD-Studie wurde der Wissensstand zu den STI auf verschiedene Weise erfasst. Wir fragten zunächst: „Welche sexuell übertragbaren Infektionen (STI)/Erkrankungen kennen Sie?“, und baten alle Befragten, die ihnen bekannten Infektionen/Erkrankungen in ein offenes Textfeld einzutragen. Diese „ungestützte Abfrage“ gibt Aufschluss darüber, welche STI den Befragten tatsächlich präsent und spontan benennbar sind. In einem zweiten Schritt wurde allen Teilnehmenden eine Liste vorgelegt und sie sollten angeben, von welchen der STI auf dieser Liste sie schon einmal gehört oder gelesen hatten („gestützte Abfrage“). Diese Liste war bei der ungestützten Abfrage noch nicht sichtbar. Des Weiteren fragten wir nach der subjektiven Zufriedenheit mit dem persönlichen Informationsstand zu HIV/Aids („Wie gut fühlen Sie sich über HIV/Aids informiert?“) und zu anderen STI („Wie gut fühlen Sie sich über andere STI/Erkrankungen informiert?“). Die Antwortmöglichkeiten folgten Schulnoten und reichten von *1* = „sehr gut“ bis *6* = „ungenügend“.

Die Frage nach der deutschen Muttersprache („Ist Deutsch Ihre Muttersprache?“, mit den Antwortmöglichkeiten „Ja“/„Nein“) diente als einfacher Indikator für eine familiäre Migrationsgeschichte.

Die regionale soziale Benachteiligung als externes Kriterium wurde über den sogenannten Deprivationsindex gemessen, der dem erhobenen Datensatz über eine regionale Kennung (Postleitzahlen) hinzugefügt wurde. Der Deprivationsindex (German Index of Socioeconomic Deprivation – GISD) basiert auf den Daten zu Beruf, Bildung und Einkommen der Wohnbevölkerung von Regionen und bildet entsprechend regionale sozioökonomische Unterschiede als Maß für soziale Benachteiligung ab. Er ist ein Merkmal der Wohnregion, nicht ein Merkmal der Person und daher geeignet, regionale Unterschiede in der Gesundheit zu erklären. Der Index wird in der Forschung und Gesundheitsberichterstattung des Bundes und der Länder genutzt und soll dazu beitragen, neue Datenquellen für die Analyse des Zusammenhangs von sozialer Ungleichheit und Gesundheit zu erschließen. In der Aufschlüsselung nach Quintilen bedeutet das 1. Quintil „niedrige Deprivation“ und das 5. Quintil „höchste Deprivation“ [23].

### Statistische Auswertung

Die statistische Berechnung erfolgte auf Basis des nach dem Mikrozensus 2018 gewichteten Datensatzes. Die Auswertung der stratifizierten und geclusterten Stichprobe wurde mit dem Modul „Complex Samples“ der Datenanalysesoftware IBM SPSS Statistics 22.0. durchgeführt. Prävalenzen der STI-Kenntnisse mit zugehörigem 95 %-Konfidenzintervall wurden mit den gewichteten Daten berechnet und geschlechts- und altersspezifisch berichtet. Für Unterschiede in den Kenntnissen zwischen Männern und Frauen bzw. den Altersgruppen wurden Chi-Quadrat-Tests in der Version für komplexe Stichproben berechnet, das Signifikanzniveau wurde mit 0,05 gewählt. Zur Bestimmung weiterer das STI-Wissen beeinflussender Faktoren wurde entlang der Anzahl der spontanen Nennungen in zwei Gruppen unterteilt: „gut“ = spontane Nennung von drei oder mehr STI, „gering“ = keine, eine oder zwei STI konnten spontan genannt werden. Diese Variable wurde in einer logistischen Regressionsanalyse als Outcome genutzt. Als erklärende Variablen wurden die soziodemografischen Faktoren Alter, Bildung, Muttersprache und Deprivation des Wohnortes sowie die sexualitätsbezogenen Faktoren Anzahl der SexualpartnerInnen, Lebenszeitprävalenz von STI und sexuelle Orientierung in die Analysen mit einbezogen.

## Ergebnisse

Verschiedene soziodemografische und sexualbezogene Basisvariablen werden zur Charakterisierung der befragten Population in Tab. 1 dargestellt. 12,9 % der Befragten waren schon einmal an einer der hier untersuchten 9 STI erkrankt (Lebenszeitprävalenz). Die Stichprobe ist vorwiegend heterosexuell (96,6 % bezeichnen die eigene sexuelle Identität als ausschließlich oder vorwiegend heterosexuell) und die überwiegende Mehrheit der Befragten (75,8 %) lebt zum Zeitpunkt der Befragung in einer festen Beziehung oder Ehe.

**Table 1:** 

	% (Gewichtetes *n*; ungewichtetes *n*)
*Alter*	
18–25	12,0 (596; 766)
26–35	17,6 (873; 1103)
36–45	16,4 (811; 816)
46–55	21,8 (1081; 870)
56–65	18,9 (934; 874)
66–75	13,3 (660; 526)
*Geschlecht*	
Männer	50,2 (2487; 2336)
Frauen	49,8 (2468; 2619)
*In fester Beziehung/verheiratet*	
Ja	75,8 (3756; 3779)
Nein	24,2 (1199; 1176)
*Bildung*	
Niedrige Bildung	31,8 (1578; 1016)
Mittlere Bildung	31,8 (1575; 1555)
Höhere Bildung	36,4 (1802; 2384)
*Sexuelle Orientierung*	
Heterosexuell^a^	96,6 (4563; 4601)
LSBA (lesbisch, schwul, bisexuell, asexuell)	3,4 (162; 179)
*Jemals an einer der hier untersuchten STI erkrankt*	
Ja	12,9 (483; 508)
Nein	87,1 (3264; 3140)

Die Prozentangaben beziehen sich auf die gewichteten Daten

^a^ausschließlich/vorwiegend

### Bekanntheit von STI

Von den neun hier untersuchten STI war HIV/Aids den meisten Befragten bekannt: 71,1 % aller Befragten konnten HIV/Aids in der ungestützten Abfrage nennen, in der gestützten Abfrage stieg der Wert auf 94,3 % an (Tab. 2). In der Rangfolge der Bekanntheit in der ungestützten Abfrage waren nach HIV/Aids die beiden am häufigsten genannten STI Gonorrhö/Tripper (38,6 %) und Syphilis (31,9 %). Die drei am wenigsten bekannten STI in der ungestützten Abfrage waren Trichomoniasis (0,4 %), Filzläuse (2,8 %) und Genitalwarzen (4,4 %).

**Table 2:** 

	Ungestützte Abfrage		*p*-Wert	Gesamt	Gestützte Abfrage		*p*-Wert	Gesamt
	Männer	Frauen			Männer	Frauen		
HIV/Aids	71,1 [68,4–73,7]	71,1 [68,6–73,4]	0,982	71,1 [69,3–72,8]	93,9 [92,4–95,1]	94,8 [93,5–95,8]	0,311	94,3 [93,3–95,1]
Syphilis	31,6 [29,3–33,9]	32,3 [29,9–34,8]	0,651	31,9 [30,2–33,7]	81,8 [79,8–83,6]	81,0 [78,9–83,0]	0,534	81,4 [79,8–82,9]
Gonorrhö/Tripper	43,9 [41,1–46,7]	33,3 [31,1–35,5]	0,000	38,6 [36,7–40,5]	73,7 [74,1–75,8]	76,0 [73,8–78,0]	0,157	74,8 [73,3–76,2]
Hepatitis B	10,5 [9,0–12,3]	10,0 [8,8–11,4]	0,644	10,3 [9,3–11,3]	69,2 [66,6–71,6]	74,1 [71,8–76,3]	0,003	71,6 [69,8–73,4]
Genitaler Herpes	9,7 [8,5–11,1]	11,5 [10,2–13,0]	0,053	10,6 [9,7–11,6]	53,3 [51,0–55,7]	60,2 [57,7–62,6]	0,000	56,8 [55,0–58,5]
Schamhaarläuse/Filzläuse	2,7 [2,1–3,6]	2,8 [2,1–3,7]	0,962	2,8 [2,3–3,4]	52,3 [49,6–54,9]	53,7 [51,2–56,2]	0,398	53,0 [51,0–54,9]
Chlamydien	7,8 [6,6–9,1]	15,6 [13,9–17,5]	0,000	11,7 [10,5–13,0]	39,5 [37,1–42,1]	60,1 [57,2–62,9]	0,000	49,8 [47,7–51,8]
Genitalwarzen	3,8 [3,0–4,8]	4,9 [4,1–6,0]	0,072	4,4 [3,8–5,1]	33,7 [31,5–35,9]	45,9 [43,6–48,2]	0,000	39,7 [38,1–41,3]
Trichomoniasis	0,2 [0,1–0,5]	0,6 [0,3–1,1]	0,043	0,4 [0,3–0,6]	7,7 [6,6–9,0]	17,6 [15,9–19,4]	0,000	12,6 [11,6–13,7]

Die Zahlen in Klammern [...] bezeichnen 95 %-Konfidenzintervalle

Geschlechterunterschiede in Bezug auf die Bekanntheit fanden sich in der ungestützten Abfrage bei Chlamydien: diese waren Frauen (15,6 %) deutlich häufiger bekannt als Männern (7,8 %), und Gonorrhö: Diese war Männern (43,9 %) häufiger bekannt als Frauen (33,3 %). In der gestützten Abfrage erkannten allerdings Frauen deutlich mehr STI und waren über Chlamydien, Hepatitis B, Genitalwarzen und Genitalherpes signifikant besser informiert als Männer (Tab. 2). Insgesamt variierte die Bekanntheit der verschiedenen STI stark. Es fällt auf, wie deutlich der Unterschied zwischen aktivem (ungestützte Abfrage) und passivem (gestützte Abfrage) Wissensstand ist.

### Bekanntheit von STI in verschiedenen Altersgruppen

**Figure 1:**
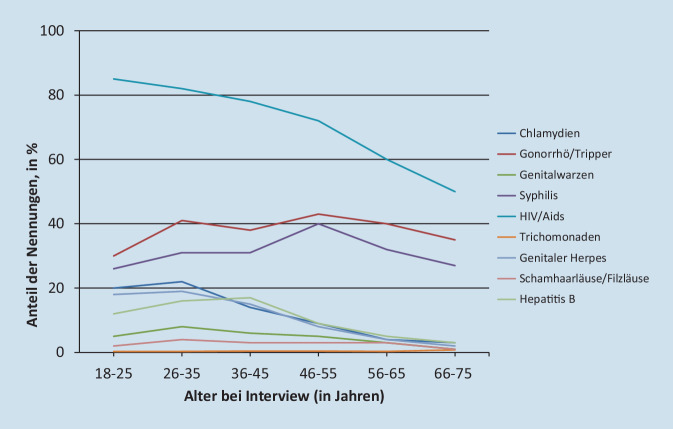

Die Informiertheit über STI unterschied sich in den Altersgruppen der Befragten deutlich (Abb. 1): Grundsätzlich zeigten sich die jüngeren Altersgruppen (18- bis 35-Jährige) deutlich besser informiert als ältere Menschen (36- bis 75-Jährige). Eine Ausnahme bildeten Syphilis und Gonorrhö/Tripper; hier stieg der Informationsstand bis zur Altersgruppe der 46- bis 55-Jährigen an und war nur bei den beiden ältesten Altersgruppen (Personen ab 56 Jahren) niedriger.

### Der Einfluss soziodemografischer und sexualitätsbezogener Merkmale auf das Wissen über STI

**Figure 2:**
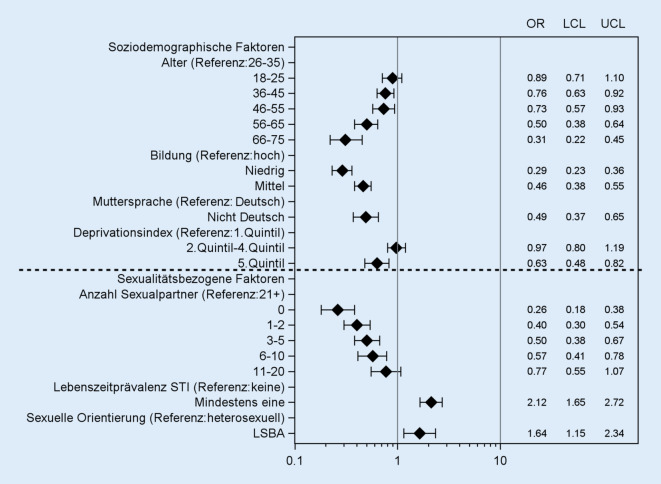

In der Regressionsanalyse trugen alle dargestellten soziodemografischen und sexualitätsbezogenen Faktoren signifikant zur Erklärung des STI-Wissensstandes bei (Abb. 2). Das Wissen über STI ist bildungsabhängig: Personen mit niedriger Bildung konnten seltener mindestens drei STI spontan nennen (Odds Ratio [OR] = 0,29). Des Weiteren war die Chance, drei und mehr STI spontan benennen zu können, deutlich niedriger bei älteren Personen (mit dem niedrigsten OR) in der Gruppe der 66- bis 75-Jährigen (OR = 0,31) sowie für Personen, die Deutsch nicht als Muttersprache erlernt haben (OR = 0,49). Ebenfalls zeigten sich geringe Chancen, drei oder mehr STI zu kennen, für sozial benachteiligte Personen, die in deprivierten Regionen leben (OR = 0,63).

Bei den sexualitätsbezogenen Faktoren hatte die Anzahl der SexualpartnerInnen den stärksten Effekt: Personen ohne bisherige Sexualkontakte hatten die geringste Wahrscheinlichkeit, drei oder mehr STI spontan nennen zu können (OR = 0,26). Mit zunehmender Anzahl der SexualpartnerInnen stieg die Chance, mindestens drei STI nennen zu können, deutlich an. Personen, die sich selber nicht als heterosexuell bezeichneten (LSBA, lesbisch, schwul, bisexuell, asexuell; OR = 1,64), wiesen ebenfalls eine erhöhte Chance auf, mindestens drei STI spontan nennen zu können. Auch Personen, die bereits an einer der hier untersuchten neun STI erkrankt waren (Lebenszeitprävalenz, OR = 2,12), zeigten sich signifikant besser informiert.

### Subjektive Bewertung des Informationsstandes zu verschiedenen STI

Im Hinblick auf die subjektive Bewertung des Informationsstandes zeigte sich im Vergleich von HIV/Aids und anderen STI, dass sich das eingangs schon erwähnte Informationsgefälle auch in der subjektiven Einschätzung der Befragten abbildet: Während zwischen 62,5 % (18- bis 25-Jährige) und 79,9 % (46- bis 55-Jährige) aller Befragten den eigenen Informationsstand bezogen auf HIV/Aids als gut oder sehr gut beurteilten, war dies bezogen auf die anderen STI nur bei 24,3 % (18- bis 25-Jährige) bzw. 41,0 % (66- bis 75-Jährige) der Befragten der Fall (Tab. 3).

**Table 3:** 

Alter (in Jahren)	Informationsstand zu sehr gut/gut	
	HIV/Aids gesamt	STI gesamt
18–25	62,5 [58,9–66,0]	24,3 [20,9–28,2]
26–35	68,6 [65,2–71,8]	30,7 [27,7–33,9]
36–45	73,7 [69,8–77,3]	32,7 [28,6–37,1]
46–55	79,9 [76,6–82,7]	36,2 [32,5–40,0]
56–65	72,2 [68,6–75,6]	38,4 [34,7–42,3]
66–75	62,5 [57,3–67,4]	41,0 [36,4–45,9]

Die Zahlen in Klammern [...] bezeichnen 95 %-Konfidenzintervalle

Tab. 3 zeigt, dass der Anteil derjenigen, die sich gut und sehr gut über STI informiert fühlten, mit zunehmendem Alter ansteigt. Diese subjektive Einschätzung steht im Widerspruch zu den objektiv gemessenen Daten, die zeigten, dass die Bekanntheit der unterschiedlichen STI in den höheren Altersgruppen deutlich niedriger war (vgl. Abb. 1).

## Diskussion

Die Ergebnisse der GeSiD-Studie zeigen, dass HIV/Aids der überwiegenden Mehrheit der Befragten bekannt war und die Mehrheit den eigenen Wissensstand hierzu als gut oder sehr gut beurteilte. Die eingangs formulierte Vermutung, dass die Kenntnis von HIV/Aids sehr viel ausgeprägter ist als die von anderen STI, bestätigte sich sowohl in der ungestützten wie auch in der gestützten Abfrage. Hier zeigt sich unter anderem der große Erfolg der Ende der 1980er-Jahre gestarteten nationalen Kampagne „Gib AIDS keine Chance“ bzw. deren Folgekampagne LIEBESLEBEN, in deren Rahmen es gelungen ist, die Bevölkerung umfassend über HIV/Aids und die damit zusammenhängenden Infektionsrisiken und Schutzmaßnahmen zu informieren.

Unsere Ergebnisse zeigen aber auch, dass andere STI – von denen die meisten sehr viel häufiger vorkommen als HIV – vielen Erwachsenen nicht bekannt sind. Auch wenn wir eine große Heterogenität im Wissensstand bezogen auf die jeweils spezifischen STI finden, zeigt sich insgesamt doch, dass die Bekanntheit und das Wissen über andere STI wenig zufriedenstellend sind. Hier muss kritisch angemerkt werden, dass die bisherigen Kampagnen der BZgA offensichtlich noch nicht zu einer wesentlichen Verbesserung des Wissensstands zu STI in der Bevölkerung geführt haben. Dies spiegelt sich auch in der subjektiven Einschätzung der Befragten.

Wenig informiert zeigten sich ältere Menschen und Befragte mit niedrigem Bildungsstand. Die regionale soziale Deprivation sowie eine familiäre Migrationsgeschichte wirkten sich ebenfalls nachteilig auf das Wissen über STI aus.

Weitere Studien in Deutschland konnten einen deutlichen Einfluss von geringer Bildung und sozialer Benachteiligung auch auf andere sexuelle Gesundheitsrisiken aufzeigen: So konnte beispielsweise gezeigt werden, dass geringe Bildung und soziale Benachteiligung das Risiko, ungewollt schwanger zu werden, stark erhöhen. Das gilt sowohl für Schwangerschaften von minderjährigen [24] als auch von erwachsenen Frauen [25]. Dieses ist im Sinne gleicher sexueller und reproduktiver Rechte für alle Menschen ein Ergebnis, das die Notwendigkeit der Entwicklung besonderer zielgruppenspezifischer Aufklärungsangebote unterstreicht.

Im Hinblick auf den Einfluss des Alters kann positiv vermerkt werden, dass bei den jüngeren Befragten (18–35 Jahre) die Bekanntheit der verschiedenen STI höher und der Wissensstand insgesamt besser ist als bei den älteren Geburtsjahrgängen. Dies hängt sicherlich damit zusammen, dass für die jüngeren Jahrgänge einerseits sexuelle Bildungs- und Aufklärungsangebote in der Schule generell weiterverbreitet waren und andererseits in deren Rahmen auch häufiger über STI gesprochen wurde [18].

Unsere Analysen zeigen, dass es besonders bei älteren Menschen eine Diskrepanz zwischen dem objektiv gemessenen Informationsstand und dem subjektiven Gefühl, gut informiert zu sein, gibt. Ältere Menschen, die eventuell nicht mehr sexuell aktiv sind oder keine wechselnden SexualpartnerInnen haben, fühlen sich ausreichend informiert, auch wenn sie objektiv betrachtet einen geringeren Kenntnisstand haben. Jüngere Menschen, die häufiger wechselnde SexualpartnerInnen und ein höheres Risiko einer STI haben, haben zwar einen objektiv besseren Wissensstand, fühlen sich aber dennoch weniger oft gut genug informiert.

Ebenfalls einen deutlichen Einfluss auf den Wissensstand zu STI haben sexualitätsbezogene Merkmale. Menschen mit vielen wechselnden SexualpartnerInnen (vorrangig Männer, die Sex mit Männern haben, MSM) sowie Personen, die ihre sexuelle Orientierung nicht als heterosexuell bezeichnen (LSBA), hatten den besten Wissensstand. Für beide Gruppen ist angesichts hoher PartnerInnenzahlen das statistische Risiko, sich mit einer STI anzustecken, besonders hoch und daher ein guter Kenntnis- und Informationsstand besonders wichtig. Dieses Ergebnis lässt sich dahin gehend interpretieren, dass eine höhere Exposition gegenüber sexuellen Gesundheitsrisiken mit einem höheren Informationsbedarf und gesteigertem Interesse an solchen Informationen einhergeht. Offensichtlich ist jedoch in der heterosexuellen Bevölkerung das Bewusstsein wenig verbreitet, dass STI für alle sexuell aktiven Personen ein wichtiges Thema sind.

Dieser Wissensmangel innerhalb der heterosexuellen Bevölkerung könnte die Motivation zur Verwendung von Kondomen verringern und auch den Zugang zu anderen Methoden der STI-Prävention erschweren. Dies betrifft sowohl primäre (wie z. B. Hepatitis-B- und HPV-Impfung) als auch sekundäre (wie z. B. Chlamydienscreening) Prävention. Wenn sexuell aktive Erwachsene eine informierte und fundierte Entscheidung über die Inanspruchnahme präventiver Optionen bezogen auf ihre sexuelle Gesundheit treffen sollen, müssen in einem ersten Schritt der Grad der Bekanntheit und das Wissen über STI deutlich verbessert werden. Dies ist nicht nur eine Aufgabe für verbesserte Aufklärungskampagnen, sondern fordert auch eine kritische Betrachtung der Rolle der ÄrztInnen. Diese sind in vielerlei Hinsicht die ersten AnsprechpartnerInnen, aber im klinischen Alltag fehlt oft die Zeit für die Beratung.

### Limitationen

Mit der GeSiD-Studie ist es erstmalig für die Bundesrepublik Deutschland gelungen, einen repräsentativen Sex-Survey mit Daten der erwachsenen Allgemeinbevölkerung durchzuführen. Die Stärken der Studie liegen in einer qualitativ hochwertigen Methodologie (Face-to-Face-Interviews und Einwohnermeldeamtsstichprobe) sowie einer breiten Perspektive auf sexuelle Gesundheit, die nicht nur die Verhinderung von Krankheiten und sexuellen Gesundheitsrisiken fokussiert, sondern auch Themen wie Einstellungen zur Sexualität, Online Sexual Activities, sexuelle Lust und Masturbation umfasst. Zu den Limitationen der Studie gehört die Teilnahmequote von 30,2 %, die für Deutschland zwar eine gute Ausschöpfung darstellt, aber trotzdem einem hohen Anteil von Verweigerern beinhaltet. Zum Vergleich: Die letzte Welle des European Social Survey (ESS 2019) erreichte eine Teilnahmequote von 27,6 % [26] und die Allgemeine Bevölkerungsumfrage der Sozialwissenschaften (ALLBUS), die seit 1980 repliziert wird, erzielte 2018 eine Teilnahmequote von 32,4 % [27]. Trotzdem können wir mit dieser Teilnahmequote eine Verzerrung der Ergebnisse nicht ausschließen. Es ist zu vermuten, dass sich Menschen mit liberalen Einstellungen zur Sexualität häufiger beteiligt haben. Allerdings zeigt der Vergleich der erhobenen Daten mit den Daten des Mikrozensus von 2018, dass die Erhebung eine gute Repräsentativität bezogen auf wichtige soziodemografische Basisdaten erreicht hat. Dies bestätigt sich auch in der von uns durchgeführten Verweigererbefragung^5^. Nur Personen, die ihren allgemeinen Gesundheitszustand als schlecht einschätzen, sind in der GeSiD-Stichprobe leicht unterrepräsentiert [22].

Eine weitere wichtige Einschränkung liegt in der Tatsache begründet, dass aus pragmatischen und Kostengründen keine objektiven Daten zu aktuellen oder früheren Erkrankungen mit sexuell übertragbaren Infektionen durch Urin- oder Bluttests erhoben werden konnten und die Datenanalysen daher auf selbstberichtete Informationen über die Prävalenz bestimmter STI beschränkt sind.

## Fazit

Die GeSiD-Studie zu „Gesundheit und Sexualität in Deutschland“ zeigt anhand repräsentativer Daten, dass Erwachsene in Deutschland über alle Altersgruppen hinweg lediglich über einen geringen Wissensstand zu den verbreitetsten STI verfügen – und das in einer Situation, in der die Prävalenzen und Inzidenzen einiger dieser Erkrankungen zunehmen. Es ist zu befürchten, dass die zur Verfügung stehenden wirksamen Instrumente der Primär- und Sekundärprävention deshalb oftmals nicht in Anspruch genommen werden. Wissen ist eine notwendige Voraussetzung, um eine informierte Entscheidung bezüglich der STI-Prävention, des Schutzes vor einer STI in sexuellen Risikosituationen und der Behandlung im Falle einer Erkrankung treffen zu können. Daher braucht es eine große Anstrengung und viele verschiedene koordinierte Maßnahmen, um den Wissensstand in der Allgemeinbevölkerung deutlich anzuheben. Dazu gehören große, öffentlichkeitswirksame Kampagnen ebenso wie die Sensibilisierung und Fortbildung von SexualpädagogInnen und LehrerInnen. ÄrztInnen sowie Anbieter von Gesundheitsinformationen und sexualitätsbezogenen Bildungsangeboten sollten über die aktuellen Wissensdefizite unbedingt informiert sein, um entsprechende Angebote entwickeln zu können.

## Footnotes

1 Die steigenden Prävalenzzahlen der STI (vgl. https://www.dstig.de/sexuelle-gesundheit/epidemiologie.html) bei gleichzeitiger Stagnation der HIV-Infektionen haben bspw. die Bundeszentrale für gesundheitliche Aufklärung (BZgA) veranlasst, die jahrzehntelange Kampagne „Gib AIDS keine Chance“ zu hinterfragen und neu als Kampagne „Liebesleben“ auszurichten (vgl. www.liebesleben.de). Darüber hinaus wurden im Rahmen des Sirius Bundeswettbewerbs zur HIV/STI-Prävention zwischen 2008 und 2015 innovative Projekte für besonders bedarfsgerechte und Erfolg versprechende Präventionsansätze zu HIV und anderen STI ausgezeichnet (vgl. https://www.bundesgesundheitsministerium.de/presse/pressemitteilungen/2015/2015-2-quartal/sirius-2005.html).

2 Wir fragten im Rahmen der GeSiD-Studie ebenfalls zum Wissensstand bezüglich einer HPV-Impfung (beide Geschlechter) und ob eine HPV-Impfung vorgenommen wurde (nur Frauen zwischen 18 und 35 Jahren). Die entsprechenden Datenauswertungen werden an anderer Stelle publiziert.

3 Wir erfragten auch das Vorkommen und die Bekanntheit der sogenannten urogenitalen Erkrankungen wie Harnröhrenentzündung, Eileiterentzündung, Scheidenpilzinfektion, bakterielle Scheidenentzündung und bakterielle Blasenentzündung. Diese sind in der Allgemeinbevölkerung besonders bei Frauen sehr viel verbreiteter als die hier untersuchten neun STI und werden zu einem späteren Zeitpunkt gesondert analysiert.

4 Der Fragebogen kann bei der Erstautorin angefordert werden.

5 Alle Personen, die nicht an der GeSiD-Studie teilnehmen wollten, wurden gebeten, einen sehr kurzen Verweigererbogen auszufüllen. 2035 Personen haben an dieser Verweigererbefragung teilgenommen. Der Vergleich ihrer Angaben mit der Stichprobe zeigt, dass sich beide Gruppen bezogen auf wichtige soziodemografische Angaben recht ähnlich sind.
